# Zang-Si-Wei and its core constituent ellagic acid alleviate LPS-induced acute lung injury by modulating PI3K/AKT signaling and NLRP3-associated pyroptosis

**DOI:** 10.3389/fphar.2026.1966973

**Published:** 2026-10-27

**Authors:** Doudou Tang, Boyang Song, Huihui Wang, Yewei Wang, Mingjie Chen, Shangjie Wu

**Affiliations:** 1 Department of Internal Medicine, Hunan Institute for Tuberculosis Control (Hunan Chest Hospital), Changsha, China; 2 Department of Pulmonary and Critical Care Medicine, The Second Xiangya Hospital, Central South University, Changsha, China; 3 Hunan Centre for Evidence-based Medicine, Central South University, Changsha, China; 4 Research Unit of Respiratory Disease, Central South University, Changsha, China; 5 Clinical Medical Research Center for Pulmonary and Critical Care Medicine in Hunan Province, Changsha, China; 6 Diagnosis and Treatment Center of Respiratory Disease in Hunan Province, Changsha, China; 7 Department of Hematology, The Second Xiangya Hospital, Central South University, Changsha, China; 8 Shanghai NewCore Biotechnology Co., Ltd., Shanghai, China

**Keywords:** acute lung injury, ellagic acid, NLRP3 inflammasome, PI3K/AKT pathway, pyroptosis, Zang-Si-Wei (ZSW)

## Abstract

**Background:**

Acute lung injury (ALI) is a life-threatening respiratory condition characterized by uncontrolled pulmonary inflammation and epithelial barrier dysfunction. Zang-Si-Wei Qingfei Mixture (ZSW) is a promising Tibetan herbal formula used clinically to alleviate pulmonary symptoms, yet its pharmacological targets and therapeutic pathways in ALI have not been fully explored. This study aims to identify the bioactive constituents of ZSW and elucidate their protective and therapeutic mechanisms in ALI.

**Methods:**

ZSW’s components were identified via liquid chromatography-tandem mass spectrometry (LC-MS/MS) previously and network pharmacology. Target affinity was evaluated using molecular docking and molecular dynamics simulations. *In vivo*, C57BL/6J mice with lipopolysaccharide (LPS)-induced ALI were used to evaluate the pretreatment and post-treatment effects of ZSW and its core component, Ellagic Acid (EA). *In vitro*, MLE-12 cells and the PI3K/mTOR dual inhibitor BEZ235 were utilized to investigate the role of PI3K/AKT signaling and NLRP3 inflammasome activation and pyroptosis.

**Results:**

EA was identified as ZSW’s core bioactive constituent, demonstrating high binding stability with AKT1, PIK3CA, and PIK3R1. *In vivo*, ZSW and EA significantly attenuated lung histological damage, edema, and alveolar-capillary hyperpermeability. Both markedly reduced pro-inflammatory cytokines (TNF-α, IL-6 and IL-1β) in bronchoalveolar lavage fluid. Mechanistically, ZSW and EA inhibited PI3K and AKT phosphorylation and were accompanied by decreased NLRP3 inflammasome-associated proteins (decreased NLRP3, ASC, and cleaved Caspase-1) and GSDMD-mediated pyroptosis (decreased GSDMD-N and lactate dehydrogenase release). Notably, the 6-h post-LPS therapeutic model showed that both treatments effectively reversed established lung injury.

**Conclusion:**

ZSW and EA exert anti-inflammatory and lung-protective effects, accompanied by modulation of PI3K/AKT signaling and suppression of NLRP3 inflammasome-associated pyroptosis. This provides robust scientific evidence for ZSW’s clinical application and highlights EA as a promising therapeutic candidate for acute inflammatory lung diseases.

## 1 Introduction

Acute lung injury (ALI) is a severe and potentially life-threatening condition characterized by pulmonary inflammation, increased vascular permeability, and alveolar damage, leading to impaired gas exchange and respiratory failure (Long et al., 2022). It often progresses to acute respiratory distress syndrome (ARDS) if not effectively managed, with mortality rates for ALI range from 30% to 40%, and exceeding 50% in severe ARDS cases (Bellani et al., 2016; Bos and Ware, 2022). ALI is commonly caused by conditions such as sepsis, pneumonia, trauma, and inhalation of harmful substances, all of which trigger a cascade of inflammatory responses in the lungs. This response involves immune cells like neutrophils and macrophages, which release pro-inflammatory cytokines (e.g., TNF-α, IL-1β, IL-6) and chemokines, further exacerbating lung tissue damage (Wang et al., 2024). Traditional treatment strategies for ALI focus on supportive care, including mechanical ventilation and treatment of the underlying causes (Bos and Ware, 2022). Dexamethasone, a potent glucocorticoid, plays a crucial role in the treatment of ALI and ARDS, particularly when given early to reduce inflammation and improve outcomes (Yang et al., 2020). However, long-term or high-dose dexamethasone may lead to side effects like immunosuppression, increasing the risk of secondary infections, hypertension, muscle wasting, and hyperglycemia (Noreen et al., 2021). Despite advancements in critical care, the high morbidity and mortality associated with ALI highlight the need for more effective therapies.

Traditional Chinese Medicine (TCM) and ethnomedicine offer a promising reservoir of bioactive compounds for treating complex inflammatory diseases (Liu et al., 2025; Zhang et al., 2025). Unlike the “one-drug-one-target” approach, herbal formulations exert therapeutic effects through multicomponent and multitarget synergy, which is particularly suited for the heterogeneous pathophysiology of ALI (Liu et al., 2024). Among these, Tibetan medicine, with its unique theory and centuries of clinical history, has demonstrated remarkable efficacy in managing respiratory diseases.

Traditional Tibetan herbal formula *Shiwei Longdanhua*, composed of ten plant species native to the Qinghai-Tibet Plateau, has been used for centuries in Tibet to treat respiratory diseases, including acute bronchitis and chronic obstructive pulmonary disease (Wei et al., 2023). Zang-Si-Wei Qingfei Mixture (ZSW) is a modified and simplified version of this formula, comprising *Rhododendron anthopogonoides*, *Gentiana straminea*, *Corydalis yanhusuo*, and *Berberis kansuensis* (Lei and Wu, 2024). Its formulation ratios, extraction methods, preparation processes, and LC-MS/MS characterization have been previously reported (Wu et al., 2025). In clinical practice, ZSW has been shown to alleviate primary symptoms of chronic lung diseases, including dyspnea, cough, and sputum production (Yi et al., 2024). Mechanistic studies indicate that ZSW mitigates particulate matter-induced pulmonary inflammation and fibrosis by inhibiting the TGF-β/SMAD signaling pathway (Wu et al., 2025). Furthermore, ZSW alleviates ALI by modulating the ACE2/NF-κB pathway (Lei and Wu, 2024). However, due to the inherent chemical complexity of ZSW (Wu et al., 2025), identifying the core bioactive components responsible for its clinical efficacy remains a significant challenge.

In recent years, the integration of bioinformatics and network pharmacology has revolutionized the study of complex TCM formulations (Li et al., 2023; Zhao et al., 2023). By constructing “component-target-disease” networks, researchers can systematically predict the material basis and molecular mechanisms of herbal medicines. Coupled with molecular docking and molecular dynamics (MD) simulations, this approach provides a high-throughput and rigorous platform for screening core active ingredients and validating their binding stability with potential targets (Khan et al., 2024). This strategy bridges the gap between traditional wisdom and modern evidence-based pharmacology, offering a robust framework for investigating ZSW’s therapeutic potential in ALI.

In the present study, we employed a combined strategy of network pharmacology and experimental validation to explore the protective and therapeutic effects of ZSW against LPS-induced ALI. We first utilized network pharmacology analysis, molecular docking and molecular dynamics simulations to identify Ellagic Acid (EA) as a core bioactive component of ZSW and to identify the PI3K/AKT signaling pathway as a potentially relevant signaling pathway. Subsequently, we evaluated the anti-inflammatory and barrier-protective roles of ZSW and EA in *in vivo* mouse models and *in vitro* MLE-12 cell models. We further examined the effects of ZSW and EA on PI3K/AKT signaling, NLRP3 inflammasome-associated responses, and pyroptosis-related markers. Our findings provide new insights into the pharmacological basis of ZSW and suggest EA as a potential core compound for the treatment of acute pulmonary inflammatory diseases.

## 2 Materials and methods

### 2.1 Network pharmacology for mining ALI-related targets of ZSW bioactive components

#### 2.1.1 Identification of bioactive components and disease targets

Zang-Si-Wei (ZSW) was prepared according to the established laboratory protocol described previously (Lei and Wu, 2024; Wu et al., 2025). Based on our previous serum pharmacochemical analysis using liquid chromatography-tandem mass spectrometry (LC-MS/MS), 58 chemical components were identified in rat blood following intragastric administration of ZSW (Wu et al., 2025). In that study, the complete ZSW preparation, blank serum, and ZSW-containing serum were analyzed by LC-MS/MS, whereas the four constituent medicinal materials were not analyzed separately.

The bioactive components among these 58 compounds were predicted using the TCMSP database (https://old.tcmsp-e.com/tcmsp.php) (Ru et al., 2014), SwissADME platform (http://www.swissadme.ch/index.php) (Daina et al., 2017), and SwissTargetPrediction (http://www.swisstargetprediction.ch/) (Daina et al., 2019).

ALI-related disease targets were retrieved from GeneCards (http://www.genecards.org/) (Rebhan et al., 1997), OMIM (http://www.omim.org/) (Amberger et al., 2015), Therapeutic Target Database (TTD, http://db.idrblab.net/ttd/) (Wang et al., 2020), and DisGeNET (https://www.disgenet.org/) (Pinero et al., 2021) using the keyword “acute lung injury”. For GeneCards, targets with a relevance score >13 were selected. For DisGeNET, a threshold score of ≥0.2 was applied. Potential therapeutic targets for ALI were obtained after integrating results and removing duplicates. The intersection of ZSW bioactive component targets and ALI disease targets was visualized via a Venn diagram using the SRplot platform (http://www.bioinformatics.com.cn/SRplot) (Tang et al., 2023).

#### 2.1.2 PPI network construction and hub gene identification

The overlapping targets were imported into the STRING database (http://string-db.org) (Szklarczyk et al., 2017). The species was limited to *Homo sapiens*, and a high confidence score ≥ 0.7 was set. The protein-protein interaction (PPI) network was constructed and visualized using Cytoscape (v3.8.2) (Shannon et al., 2003). Topological analysis was performed to evaluate network parameters, where node size was proportional to the degree value. Ten hub genes were identified using the CytoHubba plugin (Chin et al., 2014) based on 12 algorithms: Degree, MCC, Clustering Coefficient, DMNC, BottleNeck, MNC, Radiality, EPC, EcCentricity, Closeness, Betweenness, and Stress.

#### 2.1.3 Sankey diagram construction

The relationship between the bioactive components of ZSW and the top 10 hub genes was visualized using a Sankey diagram (http://sankeymatic.com/).

#### 2.1.4 GO and KEGG enrichment analysis

To explore the mechanism of ZSW, Gene Ontology (GO) functional annotation and Kyoto Encyclopedia of Genes and Genomes (KEGG) pathway enrichment analysis were performed using the DAVID platform (https://davidbioinformatics.nih.gov) (Huang da et al., 2009). Significance was determined using a dual threshold of p < 0.05 and FDR < 0.05. Enrichment bubble plots were generated using SRplot.

#### 2.1.5 Molecular docking

Molecular docking between the bioactive components and the 10 hub targets was performed using AutoDock 4 and AutoDock Vina 4.2. Detailed procedures followed previously published methods (Trott and Olson, 2010).

#### 2.1.6 Molecular dynamics (MD) simulation

All-atom MD simulations were performed using GROMACS 2022. The topology for the bioactive component Ellagic Acid (EA) was generated using the General AMBER Force Field (GAFF), while the protein was parameterized with the AMBER14SB force field and the TIP3P water model. Simulations were conducted under isothermal-isobaric (NPT) and periodic boundary conditions. The LINCS algorithm was used to constrain hydrogen bonds, with an integration step of 2 fs. Long-range electrostatic interactions were calculated using the Particle Mesh Ewald (PME) method with a 1.2 nm cutoff. Simulations were maintained at 298 K and 1 bar. Following 100 ps of NVT and NPT equilibration, a 100 ns production run was performed, saving conformations every 10 ps. Principal Component Analysis (PCA) was conducted using GROMACS modules and VMD software.

#### 2.1.7 Component-target-pathway network construction

A Component-target-pathway network was constructed using Cytoscape 3.8.2, where nodes represent components, targets, and pathways, and edges represent their respective interactions.

### 2.2 Animal experiments

All animal care and experimental procedures were performed in accordance with the *Guide for the Care and Use of Laboratory Animals* of the Second Xiangya Hospital, Central South University, and were approved by the Animal Ethics Committee of the Second Xiangya Hospital (Approval Nos. 20220088 and 20240499). Lipopolysaccharide (LPS), a major component of the outer membrane of Gram-negative bacteria, is widely used to induce acute lung injury (ALI) in experimental models. In the present study, LPS was used to establish the ALI model.

#### 2.2.1 Protective effects of EA on LPS-induced ALI

Twenty-eight male C57BL/6J mice were randomly divided into four groups (n = 7/group) to evaluate the protective effects of EA: (1) Control group; (2) LPS group (5 mg/kg); (3) Low-dose EA (Low-EA, 25 mg/kg) + LPS; (4) High-dose EA (High-EA, 50 mg/kg) + LPS. Doses were determined based on previous literature (Murugan et al., 2009). EA was administered via tail vein injection (i.v.) due to its poor oral bioavailability (Seeram et al., 2004; Yan et al., 2014). Groups 3 and 4 received daily i. v. Injections for 3 days; Groups 1 and 2 received saline (100 μL, i. v.). On day 4, LPS (5 mg/kg) or saline was administered via intratracheal (e.t.) instillation. Mice were sacrificed 72 h post-LPS for lung tissue and bronchoalveolar lavage fluid (BALF) collection. Five independent biological replicates per group were used for the analysis of each individual endpoint. Seven mice per group were initially included to ensure sufficient sample availability for the multiple downstream assays, particularly BALF- and tissue-based analyses.

#### 2.2.2 Modulation of PI3K/AKT pathway by ZSW and EA

Forty-two male C57BL/6J mice were randomized into six groups: (1) Control; (2) LPS (5 mg/kg); (3) ZSW + LPS (0.2 mL ZSW i.g.); (4) High-EA + LPS (50 mg/kg i. v.); (5) Dexamethasone (Dex, 1 mg/kg i. p.) + LPS; (6) BEZ235 (25 mg/kg i. p.) + LPS. ZSW was administered twice daily (i.g.) for 3 days. EA was administered once daily (i.v.) for 3 days. Dex and BEZ235 were administered via intraperitoneal (i.p.) injection 30 min before LPS challenge. Mice were sacrificed 72 h post-LPS.

#### 2.2.3 Therapeutic effects of ZSW and EA

Twenty male C57BL/6J mice were randomized into four groups (n = 5/group) to evaluate post-injury therapeutic effects. Mice were challenged with LPS (e.t., 5 mg/kg). Treatment commenced 6 h post-LPS: (1) Control; (2) LPS; (3) LPS + ZSW (0.2 mL ZSW i.g. twice daily); (4) LPS + EA (50 mg/kg i. v., once daily). Mice were sacrificed 72 h post-LPS.

#### 2.2.4 Standard procedures

Procedures for intragastric administration, tail vein/intraperitoneal injection, intratracheal instillation, BALF collection, H&E staining, and cell counting followed established laboratory protocols (Wu et al., 2025).

#### 2.2.5 Pulmonary epithelial permeability analysis

Epithelial barrier function was assessed using Evans Blue dye. Mice received i. v. Evans Blue 2 h before sacrifice. BALF was collected, and absorbance was measured at 650 nm using a UV-Vis spectrophotometer. Dye concentration (μg/mL) was calculated via a standard curve.

#### 2.2.6 Lung coefficient and wet-to-dry (W/D) ratio

Body weights were recorded before sacrifice. The lung coefficient was calculated as: (wet lung weight/body weight) × 100%. For the W/D ratio, the upper portion of the left lung was weighed (wet weight), then dehydrated at 80 °C for 72 h until a constant weight (dry weight) was achieved.

#### 2.2.7 Histopathological lung injury scoring

H&E-stained sections were independently evaluated by two experienced pathologists in a blinded manner using the Smith scoring system (Li et al., 2020). Five dimensions - exudate, capillary congestion, neutrophil infiltration, intra-alveolar hemorrhage/debris, and epithelial hyperplasia - were graded from 0 (none) to 4 (severe). Ten high-power fields (×200) per slide were randomly scored. The scores from the two pathologists were then compared. Any disagreement between the two pathologists was determined by the third independent pathologist.

#### 2.2.8 Cytokine detection

Pro-inflammatory cytokines (TNF-α, IL-6, and IL-1β) in BALF and cell supernatants were measured using ELISA kits (Elabscience, Wuhan, China) following the manufacturer’s instructions.

#### 2.2.9 Immunohistochemistry (IHC)

Lung sections were stained for p-PI3K, p-AKT, NLRP3, and ASC using specific antibodies (details in Supplementary Table S1) as previously described (Li et al., 2024).

#### 2.2.10 Reverse transcription-quantitative polymerase chain reaction (RT-qPCR)

Total RNA was extracted from lung tissues, and mRNA levels of TNF-α, IL-6, and IL-1β were measured using RT-qPCR. Primers are listed in Supplementary Table S2.

#### 2.2.11 Western blotting (WB)

Lung tissues (20 mg) were homogenized in 200 μL lysis buffer using a pre-cooled tissue grinder. Protein concentrations were determined, and WB was performed to detect PI3K (Cell Signaling Technology, #4292), p-PI3K (Cell Signaling Technology, #4228), AKT (Cell Signaling Technology, #4691), p-AKT (Cell Signaling Technology, #4060), NLRP3 (Cell Signaling Technology, #15101), GSDMD (Proteintech, 20770-1-AP), and GSDMD-N (Proteintech, 20770-1-AP) according to standard protocols (Tang et al., 2021).

### 2.3 Cellular experiments

#### 2.3.1 Cell culture and treatment

Mouse lung epithelial cells (MLE-12) were cultured in high-glucose DMEM supplemented with 10% FBS at 37 °C and 5% CO2. Groups included: (1) Control; (2) LPS (5 μg/mL); (3) LPS + ZSW (10% ZSW-containing serum); (4) LPS + EA (10 μM); (5) LPS + BEZ235 (100 nM). Cells were pretreated with ZSW or EA for 48 h before a 12-h LPS stimulation. BEZ235 was added simultaneously with LPS. The 10% concentration was selected based on our previous cell-viability assays, in which 0%–20% ZSW-containing serum did not significantly reduce A549 or MRC-5 cell viability (Wu et al., 2025).

#### 2.3.2 Protein extraction and WB

MLE-12 cells were lysed using RIPA buffer on ice after termination of treatments. WB was performed to detect levels of PI3K, p-PI3K, AKT, p-AKT, NLRP3, Caspase-1 (Proteintech, 81482-1-RR), Cleaved Caspase-1 (Cell Signaling Technology, #89332), IL-1β (Huabio, HA601002), GSDMD, and GSDMD-N (Proteintech, 20770-1-AP).

#### 2.3.3 Cytokine and LDH assay

Cell culture supernatants were collected for cytokine measurement (ELISA). Lactate dehydrogenase (LDH) release was assessed using an LDH Cytotoxicity Assay Kit (Elabscience Biotechnology) to evaluate pyroptosis.

#### 2.3.4 Immunofluorescence

Cells were seeded on coverslips in 12-well plates. After treatment, cells were fixed with 4% paraformaldehyde, permeabilized with 0.1% Triton X-100, and blocked with goat serum. Cells were incubated with primary antibodies overnight at 4 °C, followed by fluorescent secondary antibodies (Proteintech, SA00013-2, 1:500) for 1 h at room temperature. Nuclei were stained with DAPI, and images were captured using a fluorescence microscope.

### 2.4 Statistical analysis

Data normality was first assessed using the Shapiro-Wilk test. For comparisons between two groups with normal distribution, the Student’s t-test was employed; otherwise, the Mann-Whitney U test was used. Multi-group comparisons were performed using one-way analysis of variance (ANOVA) followed by Bonferroni *post hoc* testing (for normally distributed data) or the Kruskal-Wallis test with Dunn’s correction (for nonparametric data). All values are presented as the mean ± standard error of the mean (S.E.M.) obtained from at least three independent experiments. Statistical significance was defined as P < 0.05. Statistical analyses were conducted using GraphPad Prism software.

## 3 Results

### 3.1 Mining of ALI-related disease targets for bioactive components of ZSW

Based on our previous liquid chromatography-tandem mass spectrometry (LC-MS/MS) analysis, 58 components were identified in rat blood following oral administration of ZSW (Wu et al., 2025). Utilizing the TCMSP database and SwissADME platform, we identified four primary bioactive components: Palmatine, (+)-Erythraline, Ellagic Acid (EA), and 3′-Desmethylpapaverine. A total of 329 potential targets for these four components were predicted using TCMSP, SwissTargetPrediction, and PharmMapper.

By integrating ALI-related disease targets from GeneCards, OMIM, Therapeutic Target Database (TTD), and DisGeNET, we identified 1,493 unique targets after removing duplicates. The intersection of the 329 component targets and 1,493 disease targets yielded 139 potential therapeutic targets of ZSW for ALI (Figure 1A). These targets were imported into the STRING database to construct a protein-protein interaction (PPI) network, visualized via Cytoscape (Figure 1B). Applying 12 CytoHubba algorithms, we identified 10 hub genes: *EGFR, PIK3CA, PIK3CD, PIK3R1, AKT1, SRC, STAT3, HSP90AA1, ESR1,* and *ERBB2* (Figure 1C). The targeting relationships between the four ZSW components and these hub genes were illustrated using a Sankey diagram (Figure 1D).

**FIGURE 1:**
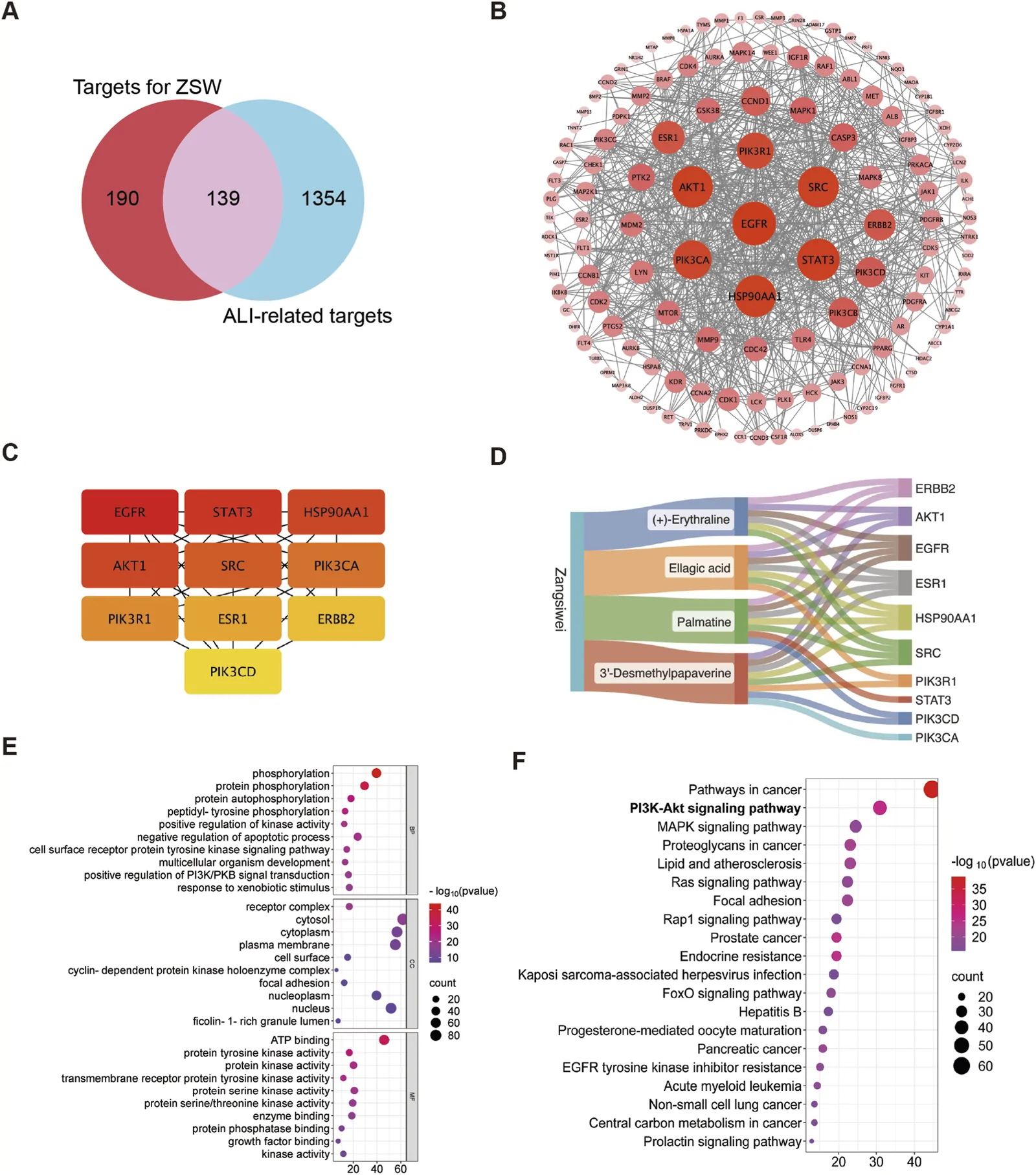
Network pharmacology analysis of the core targets and biological mechanisms of ZSW against acute lung injury. **(A)** Venn diagram illustrating the overlap between ZSW active ingredient targets and ALI disease targets. **(B)** Protein-protein interaction (PPI) network of the potential therapeutic targets constructed via the STRING database. **(C)** Identification of the top 10 hub genes evaluated by the CytoHubba Degree algorithm. **(D)** Sankey diagram delineating the targeting relationships between the four active ingredients of ZSW and the 10 hub genes. **(E)** Bubble chart displaying the top 10 significantly enriched Gene Ontology (GO) terms across biological process (BP), cellular component (CC), and molecular function (MF) categories. **(F)** Bubble chart illustrating the top 20 enriched KEGG signaling pathways. Significance thresholds were set at P < 0.05 and FDR < 0.05.

To further elucidate the mechanism of ZSW in ALI, Gene Ontology (GO) enrichment analysis was performed on the 139 potential targets (Figure 1E). The most significantly enriched terms included protein phosphorylation, positive regulation of kinase activity, and regulation of PI3K/PKB signaling. KEGG pathway analysis identified 158 significant pathways, with the PI3K/AKT signaling pathway emerging as one of the most prominent (Figure 1F), suggesting that the PI3K/AKT signaling pathway may be involved in the pharmacological effects of ZSW. Additionally, a “component-target-downstream pathway” network was constructed to delineate the multifaceted interactions of ZSW (Supplementary Figure S1).

Molecular docking was then employed to assess the binding affinity between the four ZSW components and the 10 hub proteins (Figure 2A; Supplementary Figure S2). Binding energies ranged from −6.4 to −10.3 kcal/mol, indicating stable interactions. Notably, EA exhibited a binding energy of −10.3 kcal/mol with AKT1. EA also showed strong affinity for PIK3CA and PIK3R1, key nodes in the PI3K/AKT pathway. 100 ns molecular dynamics (MD) simulations confirmed the high stability of the EA-AKT1, EA-PIK3CA, and EA-PIK3R1 complexes under physiological conditions (Figure 2B; Supplementary Figure S3). RMSD analysis showed that the EA-AKT1 complex converged to a deviation of 0.30 ± 0.05 nm after 30 ns (Figure 2B). Principal component analysis indicated a synergistic effect of EA on AKT1 and PI3K subunits (Supplementary Figure S3), while free energy landscape plots revealed a single low-energy potential well, suggesting restricted conformational rearrangement of the PI3K/AKT signaling components (Figure 2C). Binding energy calculations further corroborated the high affinity between EA and these targets (Figure 2D). Given that EA is a major constituent of ZSW and has been previously reported to alleviate hydrochloric acid-induced ALI by inhibiting inflammation (Aslan et al., 2020; Cornelio Favarin et al., 2013), these findings support EA as a potential core bioactive component of ZSW and suggest that its interaction with PI3K/AKT-related targets may contribute to the observed pharmacological effects.

**FIGURE 2:**
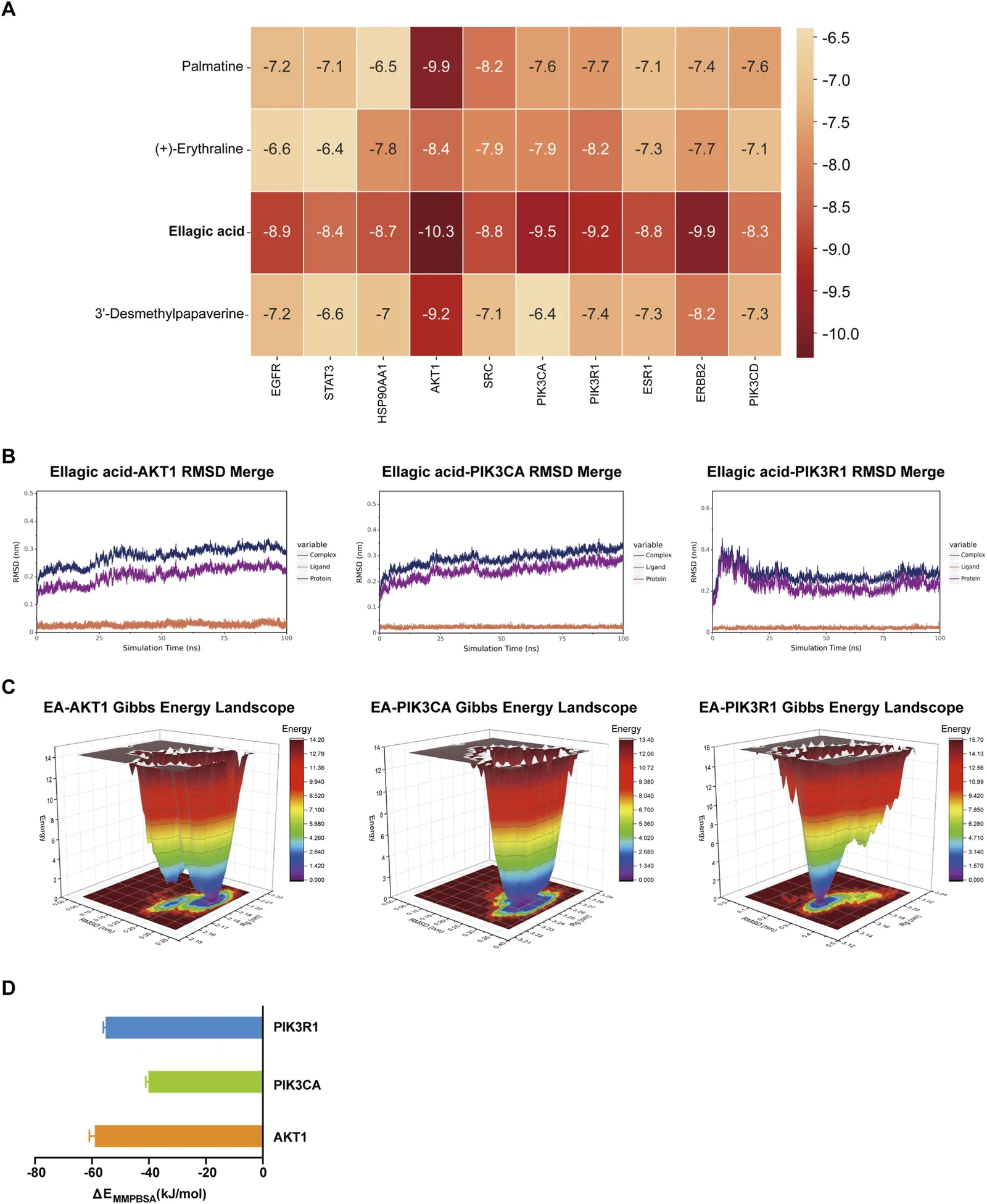
Molecular docking and molecular dynamics (MD) simulations of ZSW ingredients interacting with core targets. **(A)** Molecular docking analysis evaluating the binding affinities between the four active ingredients of ZSW and the ten potential target proteins. **(B)** Root mean square deviation (RMSD) trajectories over 100 ns MD simulations evaluating complex structural stability in a simulated physiological environment. **(C)** Free energy landscape plots revealing the low-energy basins for the respective complexes. **(D)** Binding free energy analysis of EA interacting with AKT1, PIK3CA, and PIK3R1 proteins.

### 3.2 Ellagic acid exerts protective effects against LPS-induced ALI

To validate the network pharmacology findings, we investigated whether EA could mitigate LPS-induced ALI in mice. Twenty-eight C57BL/6J mice were randomized into four groups, with pretreatment administered prior to LPS challenge. LPS exposure resulted in marked pathological changes, including pulmonary edema, alveolar wall thickening, and inflammatory cell infiltration. High-dose EA pretreatment significantly attenuated these injuries, demonstrating its anti-ALI potential (Figure 3A).

**FIGURE 3:**
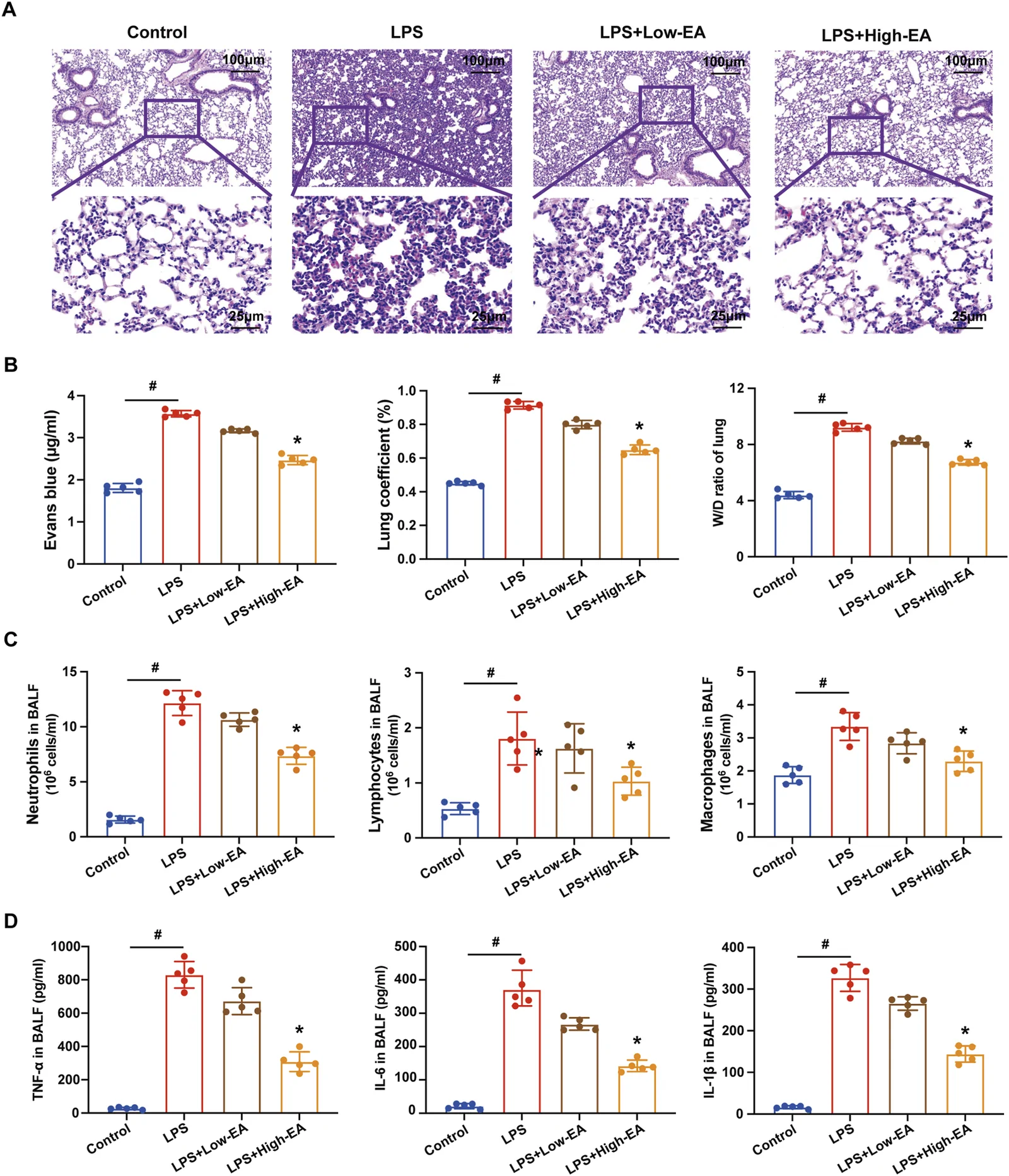
Pretreatment with ellagic acid (EA) attenuates LPS-induced acute lung injury and inflammation in mice. **(A)** Representative H&E staining images of mouse lung tissues showing pathological changes. **(B)** Quantification of Evans blue dye extravasation, lung coefficient, and lung wet-to-dry (W/D) weight ratio. **(C)** Differential inflammatory cell counts (neutrophils, lymphocytes, and macrophages) in the BALF. **(D)** ELISA analysis of pro-inflammatory cytokines (TNF-α, IL-6, and IL-1β) in the BALF. Data are presented as mean ± SEM (n = 5 independent biological replicates per group). ^#^P < 0.05 versus the control group; *P < 0.05 versus the LPS group.

A hallmark of ALI is epithelial barrier dysfunction, leading to increased permeability and edema. LPS challenge significantly increased Evans blue extravasation, the lung coefficient, and the lung wet-to-dry (W/D) weight ratio. However, high-dose EA pretreatment significantly improved these changes compared to the LPS group (Figure 3B). Differential cell counts in the bronchoalveolar lavage fluid (BALF) revealed that LPS-induced increases in neutrophils, lymphocytes, and macrophages were significantly suppressed by high-dose EA (Figure 3C). Furthermore, EA significantly inhibited the elevation of pro-inflammatory cytokines (TNF-α, IL-6, and IL-1β) in the BALF (Figure 3D). Collectively, these results indicate that high-dose EA pretreatment significantly alleviates LPS-induced lung injury, barrier dysfunction, and hyperpermeability while reducing inflammatory infiltration and cytokine secretion.

### 3.3 ZSW and EA modulate PI3K/AKT signaling in LPS-induced ALI

To further clarify the roles of ZSW, EA, and the PI3K/AKT pathway in ALI, we utilized the PI3K/mTOR dual inhibitor BEZ235. Forty-two mice were randomized into six groups. Both ZSW and high-dose EA pretreatment significantly alleviated LPS-induced lung pathology, while BEZ235 also produced protective effects, indicating that pharmacological modulation of PI3K/mTOR signaling can attenuate LPS-induced lung injury in this model (Figure 4A).

**FIGURE 4:**
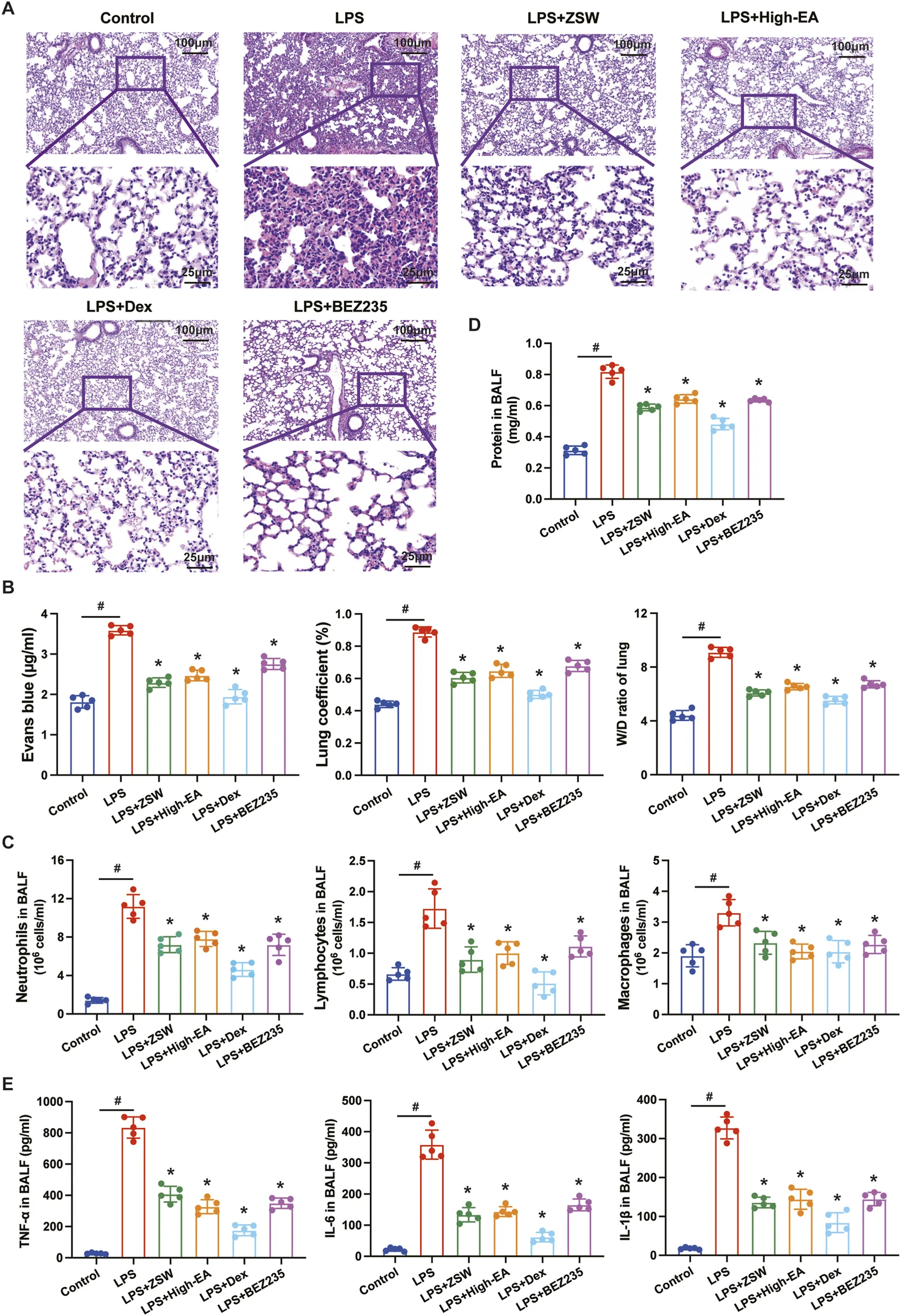
Protective effects of ZSW and EA against LPS-induced ALI in mice. Mice were randomly divided into six groups: Control, LPS, LPS + ZSW, LPS + High-EA, LPS + Dex, and LPS + BEZ235. **(A)** Representative H&E staining images of lung tissues demonstrating pathological variations among the groups. **(B)** Evaluation of alveolar epithelial permeability and pulmonary edema, including Evans blue dye extravasation, lung coefficient, and lung W/D weight ratio. **(C)** Differential inflammatory cell counts in the BALF. **(D)** Total protein concentration in the BALF indicating protein exudation. **(E)** Concentration of pro-inflammatory cytokines TNF-α, IL-6, and IL-1β in BALF. Data are presented as mean ± SEM (n = 5 independent biological replicates per group). ^#^P < 0.05 versus the control group; *P < 0.05 versus the LPS group.

Both ZSW and high-dose EA significantly improved Evans blue extravasation, lung coefficients, and W/D ratios (Figure 4B), while reducing inflammatory cell counts in the BALF (Figure 4C). Total protein concentrations (Figure 4D) and cytokine levels (Figure 4E) in the BALF were also markedly reduced by ZSW, high-dose EA, or BEZ235. Immunohistochemical (IHC) staining revealed that ZSW and EA significantly decreased the levels of phosphorylated PI3K (p-PI3K) and AKT (p-AKT) in lung tissues (Figure 5), supporting modulation of PI3K/AKT signaling by these treatments.

**FIGURE 5:**
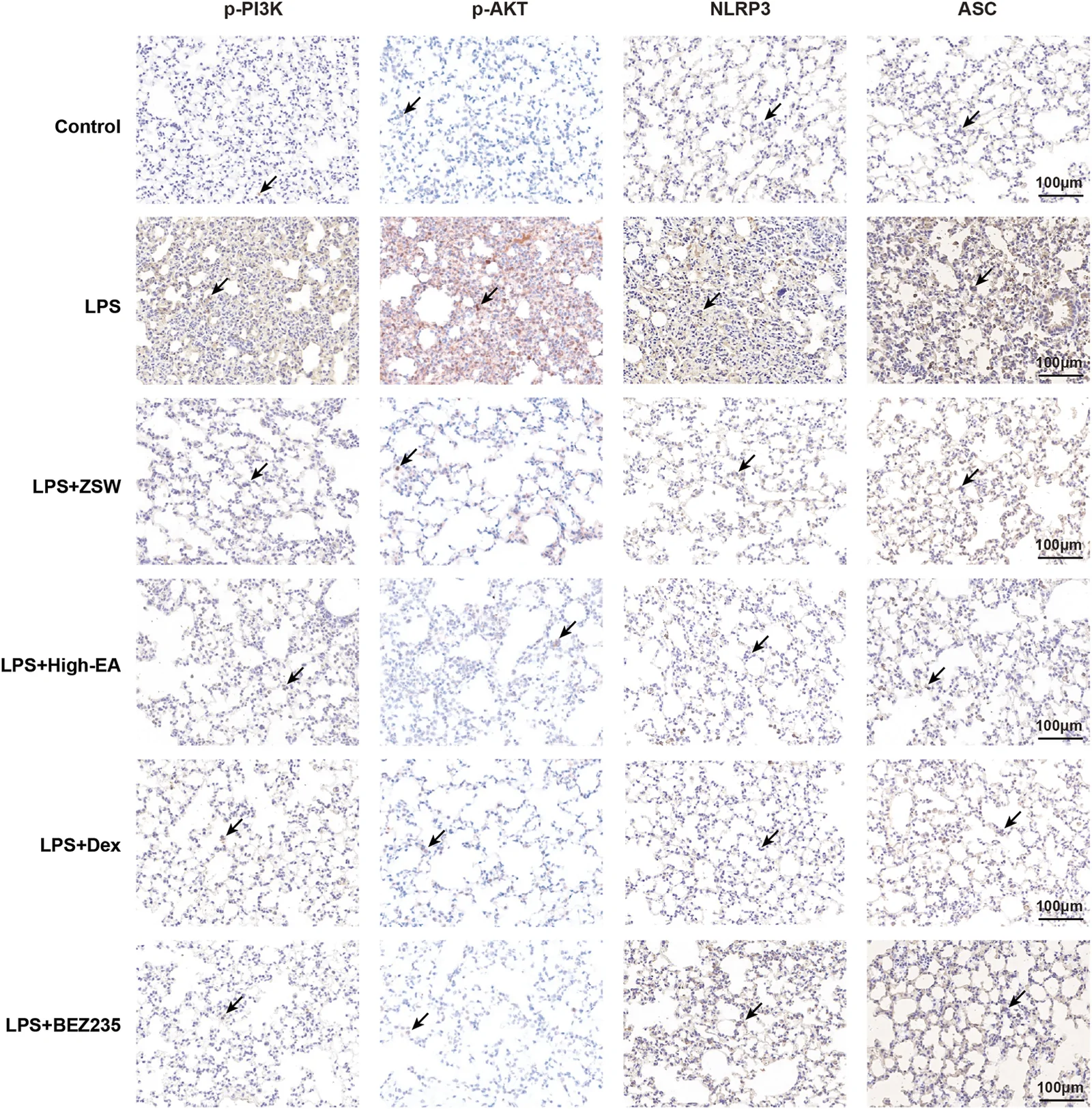
Representative immunohistochemical (IHC) staining images evaluating the expression of p-PI3K, p-AKT, NLRP3, and ASC in mouse lung tissues. Arrows indicate representative areas of positive immunoreactivity.

Given the established involvement of the NLRP3 inflammasome in inflammatory lung injury, we further examined NLRP3-associated responses. LPS exposure induced the expression of NLRP3 and the adaptor protein ASC, whereas ZSW, EA, and BEZ235 significantly inhibited their expression (Figure 5). These findings indicate that modulation of PI3K/AKT signaling and suppression of NLRP3 inflammasome-associated responses occur in parallel following treatment with ZSW or EA.

### 3.4 ZSW and EA suppress NLRP3 inflammasome-associated responses and pyroptosis in LPS-stimulated MLE-12 cells

To investigate whether these effects were accompanied by changes in NLRP3 inflammasome-associated responses and pyroptosis, we performed *in vitro* studies using mouse MLE-12 lung epithelial cells. ZSW or EA pretreatment significantly reduced the LPS-induced release of TNF-α, IL-6, and IL-1β in MLE-12 cells (Figure 6A). Consistent with *in vivo* findings, BEZ235 also inhibited cytokine release. LPS stimulation increased the phosphorylation of PI3K and AKT, an effect that was significantly reversed by ZSW and EA (Figure 6B).

**FIGURE 6:**
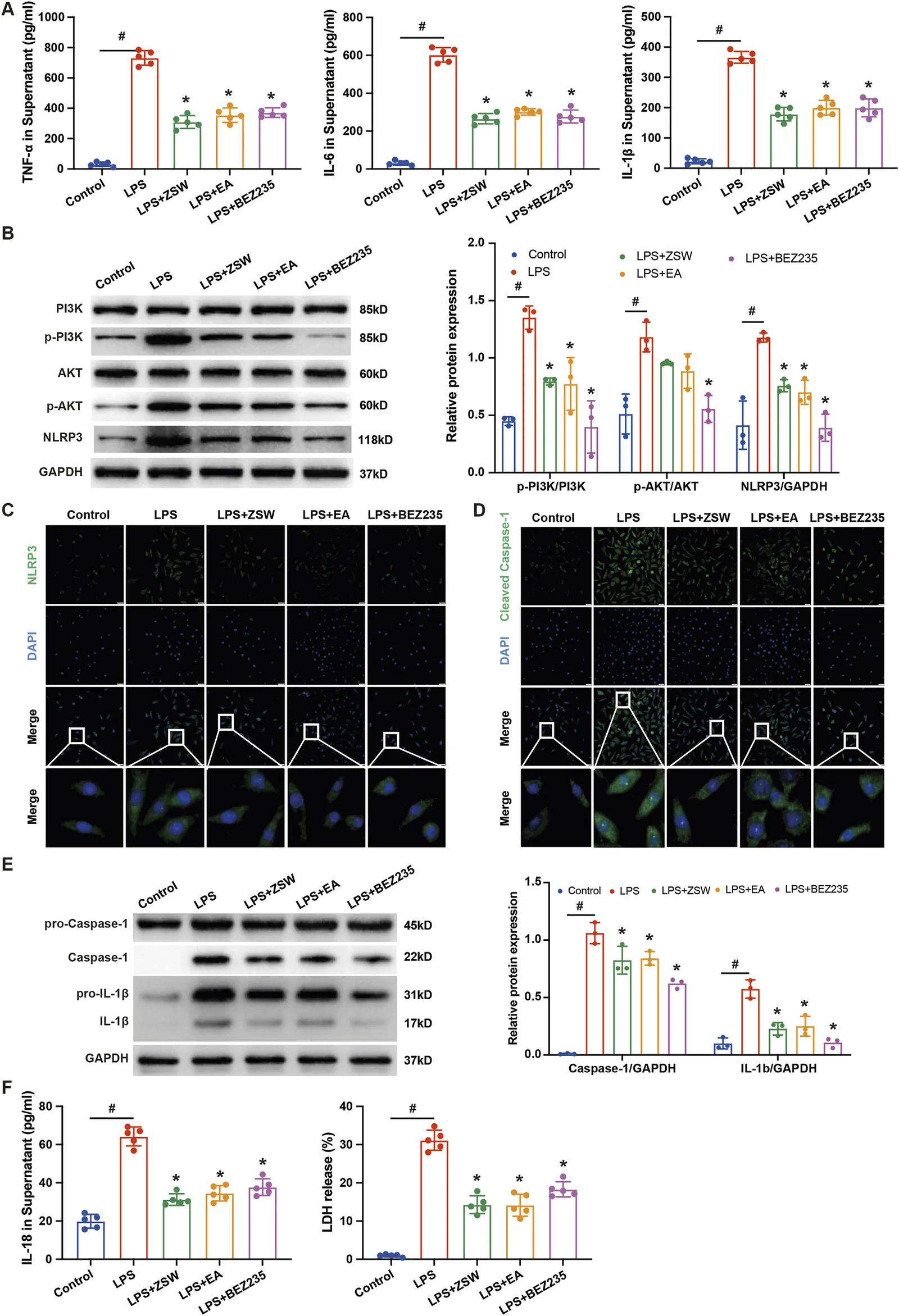
ZSW and EA attenuate LPS-induced inflammatory responses and pyroptosis-associated changes in MLE-12 cells, accompanied by modulation of PI3K/AKT signaling and suppression of NLRP3-associated responses. **(A)** Levels of TNF-α, IL-6, and IL-1β in MLE-12 cell culture supernatants measured by ELISA. **(B)** Western blot analysis of PI3K, p-PI3K, AKT, p-AKT, and NLRP3 protein expression in cell lysates. **(C)** Representative immunofluorescence images of NLRP3 expression in MLE-12 cells. **(D)** Representative immunofluorescence images showing the expression of activated Caspase-1. **(E)** Western blot analysis of pro-Caspase-1, cleaved Caspase-1 (p20), pro-IL-1β, and mature IL-1β. **(F)** Quantification of IL-18 secretion and lactate dehydrogenase (LDH) release in culture supernatants. Data are presented as mean ± SEM (n = 5 biological replicates per group). ^#^P < 0.05 versus the control group; *P < 0.05 versus the LPS group.

LPS treatment markedly induced NLRP3 expression in MLE-12 cells, which was attenuated by ZSW and EA pretreatment (Figures 6B,C). Since NLRP3 activation leads to the cleavage of pro-Caspase-1 into active Caspase-1, we measured cleaved Caspase-1 (p20) levels. LPS promoted Caspase-1 activation, while ZSW and EA significantly reduced its levels (Figures 6D,E). Furthermore, activated Caspase-1 cleaves pro-IL-1β and Gasdermin D (GSDMD). LPS-induced increases in mature IL-1β and IL-18, as well as the release of lactate dehydrogenase (LDH) - a marker of membrane rupture during pyroptosis - were all significantly reduced by ZSW and EA (Figures 6E,F). Finally, Western blot analysis showed that ZSW and EA suppressed the LPS-induced upregulation of GSDMD and the formation of the pore-forming N-terminal fragment (GSDMD-N) (Figures 7A,B). Together, these findings indicate that ZSW and EA suppress NLRP3 inflammasome-associated responses and pyroptosis-related changes in LPS-stimulated MLE-12 cells. The parallel changes in PI3K/AKT signaling and NLRP3-associated responses are consistent with involvement of both pathways in the protective effects of ZSW and EA.

**FIGURE 7:**
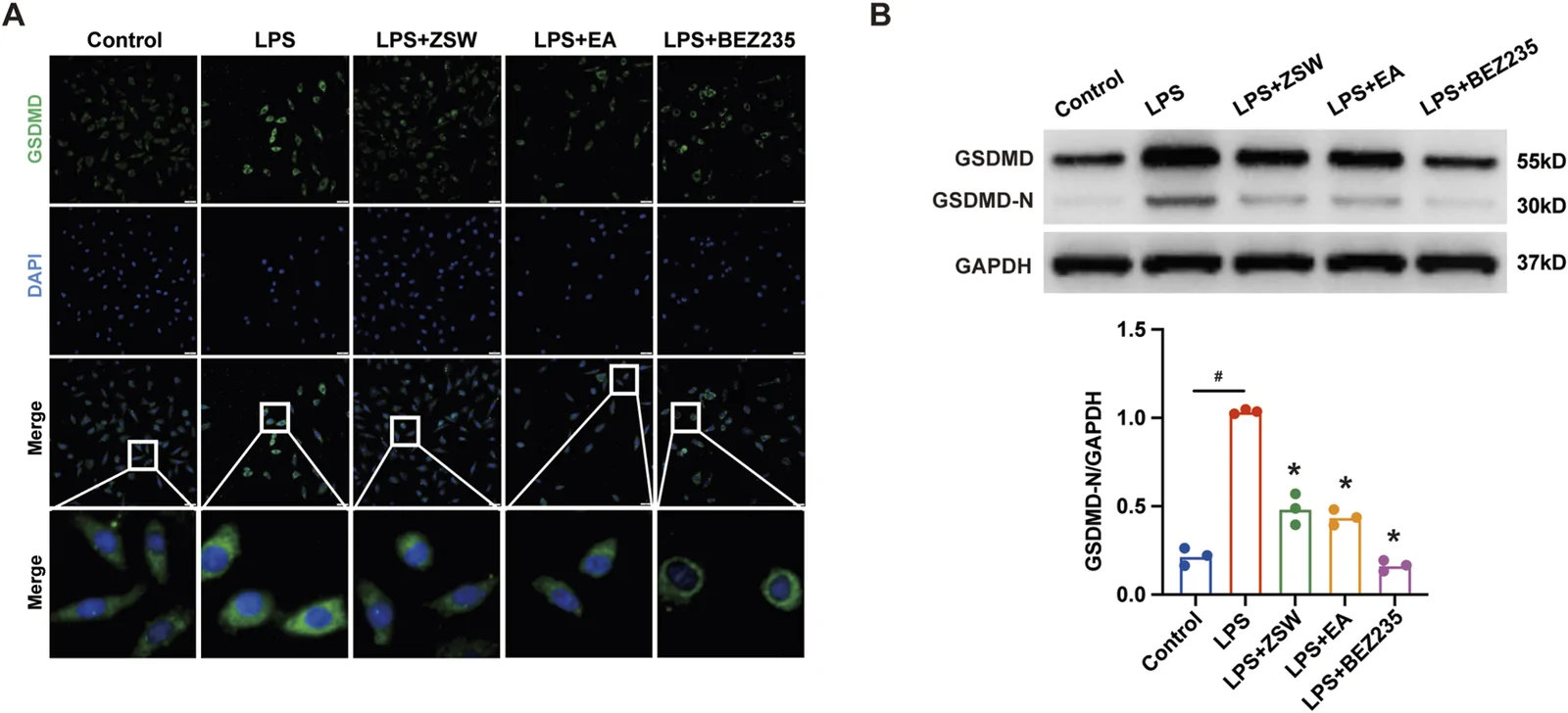
ZSW and EA attenuate GSDMD-associated pyroptosis-related changes in LPS-stimulated MLE-12 cells. **(A)** Representative immunofluorescence images illustrating the expression of GSDMD in MLE-12 cells. **(B)** Western blot analysis and corresponding quantification of full-length GSDMD and its active pore-forming N-terminal fragment (GSDMD-N) in cell lysates. Data are presented as mean ± SEM (n = 5 biological replicates per group). ^#^P < 0.05 versus the control group; *P < 0.05 versus the LPS group.

### 3.5 Therapeutic potential of ZSW and EA in LPS-induced ALI

To assess the therapeutic efficacy, we administered ZSW or EA 6 hours after LPS exposure for three consecutive days. Post-treatment with ZSW or EA significantly improved lung pathology, including edema and inflammatory infiltration (Figures 8A,B). Cell counts and protein concentrations in the BALF were also effectively reduced (Figures 8C,D). Moreover, mRNA levels of TNF-α, IL-6, and IL-1β in lung tissues were significantly downregulated following ZSW or EA treatment (Figure 8E).

**FIGURE 8:**
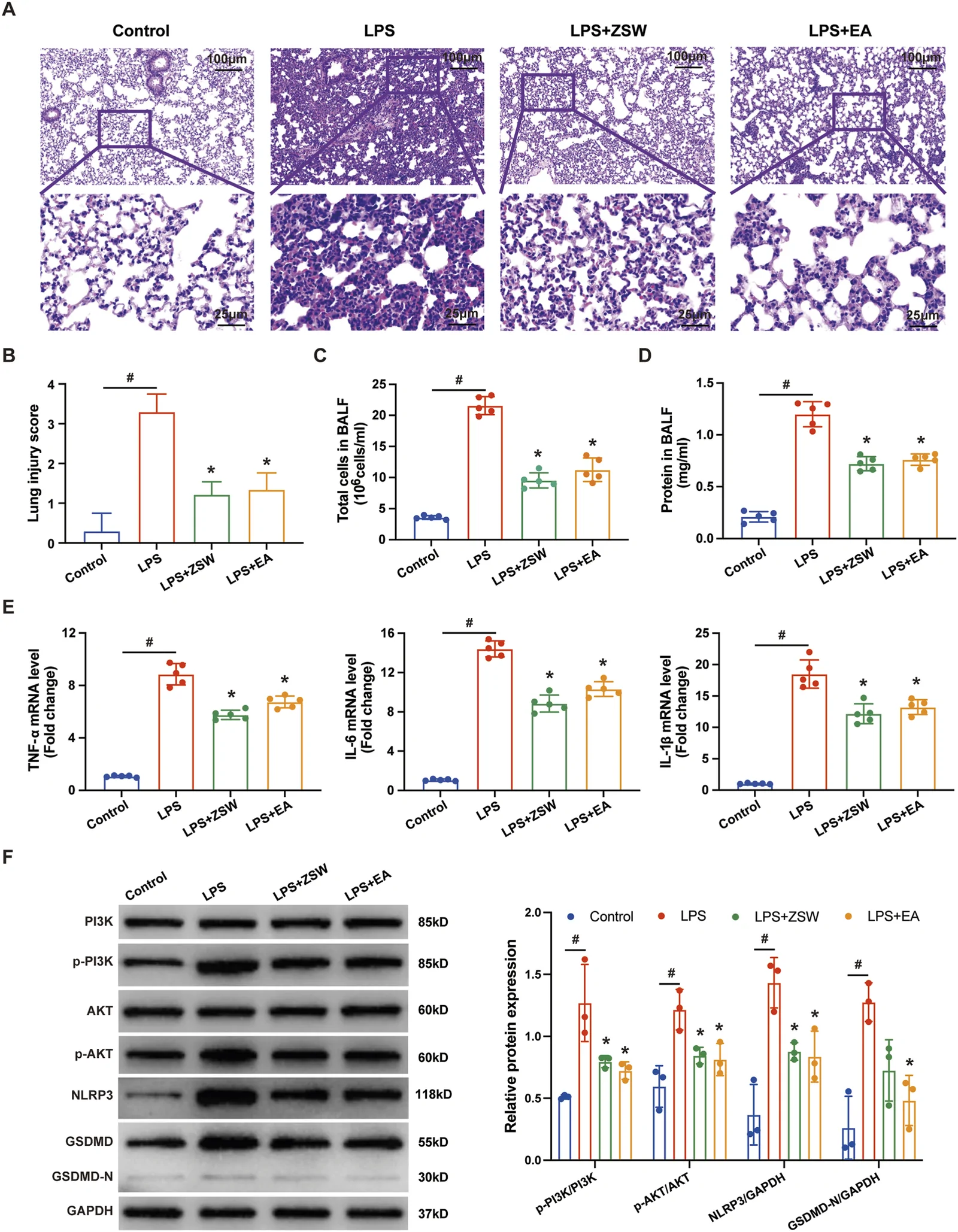
Therapeutic administration of ZSW and EA ameliorates LPS-induced ALI, accompanied by attenuation of PI3K/AKT signaling and NLRP3/pyroptosis-associated responses *in vivo*. Mice were exposed to LPS for 6 h followed by therapeutic treatment with ZSW or EA for 3 days. **(A)** Representative H&E staining images of mouse lung tissues across different groups. **(B)** Lung injury scores based on histopathological evaluation. **(C)** Total inflammatory cell counts and **(D)** total protein concentration in the BALF. **(E)** RT-qPCR analysis of pro-inflammatory cytokine (TNF-α, IL-6, and IL-1β) mRNA expression levels in lung tissues. **(F)** Representative Western blot images and quantification of PI3K, p-PI3K, AKT, p-AKT, NLRP3, GSDMD, and GSDMD-N in lung tissue homogenates. Data are presented as mean ± SEM (n = 5 per group). ^#^P < 0.05 versus the control group; *P < 0.05 versus the LPS group.

Mechanistically, Western blot analysis of lung tissues showed that ZSW and EA treatment significantly decreased the levels of p-PI3K, p-AKT, and NLRP3 (Figure 8F). Although LPS induced GSDMD expression, GSDMD-N levels did not show a significant increase in this therapeutic window. These results demonstrate that ZSW and EA possess therapeutic activity against LPS-induced ALI, accompanied by attenuation of PI3K/AKT signaling and NLRP3-associated inflammatory responses.

## 4 Discussion

Acute lung injury remains a formidable clinical challenge characterized by high mortality and limited pharmacological options. Zang-Si-Wei (ZSW), a simplified Tibetan formula, has demonstrated promising clinical efficacy in treating respiratory diseases, yet its pharmacodynamic basis and molecular mechanisms have remained largely elusive. In this study, we integrated network pharmacology, molecular docking and molecular dynamics simulations, and experimental validation to demonstrate that ZSW and its core component, Ellagic Acid (EA), alleviate LPS-induced ALI and are associated with attenuation of PI3K/AKT signaling, NLRP3 inflammasome-associated responses, and GSDMD-mediated pyroptosis.

The chemical complexity of herbal formulas often complicates the identification of their therapeutic “active center”. Through LC-MS/MS and network pharmacology, we identified EA as a primary bioactive constituent of ZSW. Our molecular docking and 100 ns molecular dynamics simulations revealed that EA possesses a high binding affinity for AKT1, PIK3CA, and PIK3R1, with binding energies significantly lower than −7.0 kcal/mol. The stability of the EA-AKT1 complex, as evidenced by converged RMSD values and a single low-energy potential well in the free energy landscape, provides a structural basis for the potential interaction of EA with PI3K/AKT-related targets and support further investigation of its effects on this signaling pathway. These findings shift the understanding of ZSW from a traditional “black box” to a more precisely defined multicomponent system where EA plays a central regulatory role. Furthermore, as a natural polyphenolic compound widely distributed in various fruits and medicinal plants, EA is well-documented in the literature for its potent anti-inflammatory effects, including the suppression of pro-inflammatory cytokine production, inhibition of neutrophil infiltration, and alleviation of epithelial barrier dysfunction (Rogerio et al., 2008; Rogerio et al., 2006).

An important novelty of the present study is the integration of serum pharmacochemistry, network pharmacology, molecular docking, and experimental validation to investigate the pharmacological basis of ZSW in LPS-induced ALI. Rather than evaluating the complex herbal preparation solely at the level of the whole formula, we first characterized circulating ZSW-derived components and subsequently identified ellagic acid (EA) as a candidate bioactive component for further investigation. The protective effects of both ZSW and EA were then consistently observed *in vivo* and in LPS-stimulated MLE-12 cells, providing complementary evidence at the organismal and cellular levels. In addition, our findings link the protective effects of ZSW and EA with attenuation of PI3K/AKT signaling, suppression of NLRP3 inflammasome-associated responses, and reduction of GSDMD-mediated pyroptosis. Furthermore, the therapeutic intervention model suggests that these beneficial effects are not limited to prophylactic treatment and may extend to therapeutic intervention. Collectively, these findings extend current knowledge by linking a complex traditional herbal formulation, together with one of its pharmacologically relevant circulating components, to convergent inflammatory and pyroptosis-associated responses in ALI.

The PI3K/AKT signaling pathway has been implicated in inflammatory responses and tissue injury in ALI (Hou et al., 2024). Hyperactivation of this pathway by LPS is associated with the recruitment of inflammatory cells and the overproduction of pro-inflammatory cytokines, including TNF-α and IL-6 (Zhou et al., 2018). Our results demonstrated that ZSW and EA significantly downregulated the phosphorylation of PI3K and AKT *in vivo* and *in vitro*, accompanied by attenuation of inflammatory responses and pulmonary injury. Pharmacological inhibition with BEZ235 also ameliorated LPS-induced lung injury and inflammatory responses. Together, these findings support the involvement of PI3K/AKT signaling in the pharmacological effects of ZSW and EA. However, because BEZ235 is a PI3K/mTOR dual inhibitor and no genetic or rescue experiment was performed, these findings should be interpreted as evidence of pathway involvement rather than definitive proof that PI3K/AKT signaling is the direct upstream regulator of the inflammatory events examined in this study. Interestingly, EA showed a comparable efficacy to Dexamethasone in reducing cytokine levels but without the systemic side effects typically associated with steroids, suggesting its potential as a candidate for further investigation in ALI.

A key finding of this study is that ZSW and EA were associated with suppression of NLRP3 inflammasome-associated responses and pyroptosis. Pyroptosis, a form of programmed inflammatory cell death mediated by Gasdermin D (GSDMD), is increasingly recognized as a contributor to lung epithelial barrier breakdown (Sun and Li, 2022; Wei et al., 2022). We found that LPS challenge markedly induced NLRP3 expression, Caspase-1 activation and GSDMD-N formation in MLE-12 cells and lung tissues. ZSW and EA attenuated these changes, accompanied by reduced levels of mature IL-1β and IL-18 and decreased LDH release. These findings support an inhibitory effect of ZSW and EA on NLRP3 inflammasome-associated pyroptotic responses. By suppressing pyroptosis, ZSW and EA not only limit the “cytokine storm” but also preserve the structural integrity of the alveolar epithelium, which is crucial for preventing pulmonary edema.

Interestingly, a discrepancy in GSDMD-N expression was observed between our *in vitro* and *in vivo* models. While LPS significantly induced both full-length GSDMD and its cleaved N-terminal fragment (GSDMD-N) in MLE-12 cells, only the upregulation of full-length GSDMD was detected in lung tissue homogenates in the therapeutic mouse model. Several factors may account for this observation. First, GSDMD cleavage is a rapid and transient biochemical event associated with acute pyroptotic execution (Balasubramanian et al., 2024; Shi et al., 2015). In our therapeutic model, lung tissues were harvested 72 h post-LPS challenge; at this late stage, the cleaved GSDMD-N fragments might have undergone proteolytic degradation or been released into the extracellular space following cell rupture, whereas the “priming” effect - evidenced by the accumulation of full-length GSDMD - remained detectable. Second, unlike the synchronized response of pure MLE-12 cell cultures, lung tissue homogenates consist of a heterogeneous population of cells (including epithelial, endothelial, and various immune cells) with different kinetic profiles of inflammasome activation. This spatial and temporal heterogeneity might dilute the detectable signal of GSDMD-N in total protein extracts. Nevertheless, the consistent suppression of full-length GSDMD by ZSW and EA across both models supports the possibility that ZSW and EA affect pyroptosis-related processes at the priming stage.

From a clinical perspective, the timing of drug administration is critical. While many studies focus on preventive pretreatment, our therapeutic model (6-h post-LPS administration) demonstrated that ZSW and EA could still significantly reverse established lung injury, reduce protein exudation in BALF, and downregulate inflammatory gene expression. This therapeutic window is highly relevant to clinical scenarios where patients often present after the initial inflammatory insult. Furthermore, although EA has historically faced challenges regarding oral bioavailability, our use of tail vein injection and the observation of ZSW’s efficacy via intragastric administration suggest that the natural matrix of the ZSW formula may enhance the delivery or synergistic activity of its components.

Despite these findings, several limitations should be acknowledged. First, although EA was identified as the core component, other constituents like Palmatine and (+)-Erythraline may also contribute to the formula’s overall efficacy through additive or synergistic effects. In addition, because the four constituent medicinal materials were not extracted and analyzed separately, the specific medicinal-material origin of each circulating component could not be experimentally determined. Second, although our pharmacological and biochemical data indicate that ZSW and EA attenuate PI3K/AKT signaling and are accompanied by suppression of NLRP3 inflammasome-associated responses, the present experimental design does not establish a direct causal relationship between these pathways. In particular, BEZ235 is a dual PI3K/mTOR inhibitor and therefore cannot be considered a pathway-specific tool for defining the PI3K/AKT-NLRP3 relationship. Further studies using genetic manipulation, pathway-specific inhibition, or rescue experiments will be required to determine whether PI3K/AKT signaling directly regulates NLRP3 activation and to clarify the precise molecular relationship between these pathways. Finally, although our findings support the therapeutic potential of ZSW and EA in LPS-induced ALI, further validation in additional experimental models and, ultimately, clinical studies will be necessary to determine their translational relevance.

In conclusion, our study provides evidence that Zang-Si-Wei Qingfei Mixture (ZSW) and its core constituent Ellagic Acid exert anti-inflammatory and lung-protective effects in LPS-induced ALI. These effects are accompanied by attenuation of PI3K/AKT signaling, suppression of NLRP3 inflammasome-associated responses, and reduction of GSDMD-mediated pyroptosis-related changes. Although the present findings support the involvement of these pathways in the pharmacological actions of ZSW and EA, the direct mechanistic relationship between PI3K/AKT signaling and NLRP3 activation remains to be established. These findings provide a pharmacological basis for further investigation of ZSW and EA as potential candidates for acute inflammatory lung diseases.

## Data Availability

The original contributions presented in the study are included in the article/Supplementary Material, further inquiries can be directed to the corresponding author.
