# Supplementary material for: Zang-Si-Wei and its core constituent ellagic acid alleviate LPS-induced acute lung injury by modulating PI3K/AKT signaling and NLRP3-associated pyroptosis
Source: Front Pharmacol. 2026 Oct 27;17:1966973. doi: 10.3389/fphar.2026.1966973 (PMC13748239; doi:10.3389/fphar.2026.1966973)
Supplement: Supplementary file 1 [file Table1.docx]

Table S1. Antibodies used for Immunohistochemistry

| Antibody | Manufacturer | Cat. No. | Dilution |
| --- | --- | --- | --- |
| p-PI3K | Cell Signaling Technology | #4228 | 1:100 |
| p-AKT | Cell Signaling Technology | #4060 | 1:100 |
| NLRP3 | Boster Biological Technology | BA3677 | 1:100 |
| ASC | Affinity | DF6304 | 1:100 |
