# Supplementary material for: Zang-Si-Wei and its core constituent ellagic acid alleviate LPS-induced acute lung injury by modulating PI3K/AKT signaling and NLRP3-associated pyroptosis
Source: Front Pharmacol. 2026 Oct 27;17:1966973. doi: 10.3389/fphar.2026.1966973 (PMC13748239; doi:10.3389/fphar.2026.1966973)
Supplement: Supplementary file 2 [file Table2.docx]

Table S2. Primers used for RT-qPCR

| Name | Sequence |
| --- | --- |
| TNF-α Forward Sequence | GGTGCCTATGTCTCAGCCTCTT |
| TNF-α Reverse Sequence | GCCATAGAACTGATGAGAGGGAG |
| IL-6 Forward Sequence | TACCACTTCACAAGTCGGAGGC |
| IL-6 Reverse Sequence | CTGCAAGTGCATCATCGTTGTTC |
| IL-1β Forward Sequence | TGGACCTTCCAGGATGAGGACA |
| IL-1β Reverse Sequence | GTTCATCTCGGAGCCTGTAGTG |
| GAPDH Forward Sequence | CATCACTGCCACCCAGAAGACTG |
| GAPDH Reverse Sequence | ATGCCAGTGAGCTTCCCGTTCAG |
